# The use of positive deviance approach to improve health service delivery and quality of care: a scoping review

**DOI:** 10.1186/s12913-024-10850-2

**Published:** 2024-04-08

**Authors:** Ayelign Mengesha Kassie, Elizabeth Eakin, Biruk Beletew Abate, Aklilu Endalamaw, Anteneh Zewdie, Eskinder Wolka, Yibeltal Assefa

**Affiliations:** 1 School of Public Health, Faculty of Medicine, The University of Queensland, Brisbane, Australia; 2 School of Nursing, College of Health Sciences, Woldia University, Woldia, Ethiopia; 3 College of Medicine and Health Sciences, Bahir Dar University, Bahir Dar, Ethiopia; 4 International Institute for Primary Health Care, Ethiopia, Addis Ababa, Ethiopia

**Keywords:** Healthcare quality, Healthcare system, Health service delivery, Positive deviance

## Abstract

**Background:**

Quality has been a persistent challenge in the healthcare system, particularly in resource-limited settings. As a result, the utilization of innovative approaches is required to help countries in their efforts to enhance the quality of healthcare. The positive deviance (PD) approach is an innovative approach that can be utilized to improve healthcare quality. The approach assumes that solutions to problems are already available within the community and identifying and sharing those solutions can help others to resolve existing issues. Therefore, this scoping review aimed to synthesize the evidence regarding the use of the PD approach in healthcare system service delivery and quality improvement programs.

**Methods:**

Articles were retrieved from six international databases. The last date for article search was June 02, 2023, and no date restriction was applied. All articles were assessed for inclusion through a title and/or abstract read. Then, articles that passed the title and abstract review were screened by reading their full texts. In case of duplication, only the full-text published articles were retained. A descriptive mapping and evidence synthesis was done to present data with the guide of the Preferred Reporting Items for Systematic Reviews and Meta-analysis extension for Scoping Reviews checklist and the results are presented in text, table, and figure formats.

**Results:**

A total of 125 articles were included in this scoping review. More than half, 66 (52.8%), of the articles were from the United States, 11(8.8%) from multinational studies, 10 (8%) from Canada, 8 (6.4%) from the United Kingdom and the remaining, 30 (24%) are from other nations around the world. The scoping review indicates that several types of study designs can be applied in utilizing the PD approach for healthcare service and quality improvement programs. However, although validated performance measures are utilized to identify positive deviants (PDs) in many of the articles, some of the selection criteria utilized by authors lack clarity and are subject to potential bias. In addition, several limitations have been mentioned in the articles including issues in operationalizing PD, focus on leaders and senior managers and limited staff involvement, bias, lack of comparison, limited setting, and issues in generalizability/transferability of results from prospects perspective. Nevertheless, the limitations identified are potentially manageable and can be contextually resolved depending on the nature of the study. Furthermore, PD has been successfully employed in healthcare service and quality improvement programs including in increasing surgical care quality, hand hygiene practice, and reducing healthcare-associated infections.

**Conclusion:**

The scoping review findings have indicated that healthcare systems have been able to enhance quality, reduce errors, and improve patient outcomes by identifying lessons from those who exhibit exceptional practices and implementing successful strategies in their practice. All the outcomes of PD-based research, however, are dependent on the first step of identifying true PDs. Hence, it is critical that PDs are identified using objective and validated measures of performance as failure to identify true PDs can subsequently lead to failure in identifying best practices for learning and dissemination to other contextually similar settings.

**Supplementary Information:**

The online version contains supplementary material available at 10.1186/s12913-024-10850-2.

## Background

The realization of universal health coverage objectives is an unattainable aspiration without the concurrent provision of healthcare services of the highest quality. For this reason, the sustainable development goal target 3.8 seeks not only financial risk protection, but also to enhance access to safe, and effective essential medications and vaccinations, and high-quality medical services [1]. To support this, international organizations including the World Health Organization (WHO) and the World Bank have been promoting the idea of universal health coverage for all people to enjoy affordable access to the entire spectrum of high-quality healthcare services they require when and where they need them, and be incorporated into the healthcare system changes in several countries around the world [2, 3].

Nevertheless, healthcare systems are grappling with a multitude of challenges when it comes to improving access and providing high-quality healthcare services on a global scale. These challenges encompass deficient data collection and monitoring systems, suboptimal organizational team culture and limited capacity, ineffective leadership, neglect of the incentivization of superior performance [4], and a dearth of evidence-based health policies to support implementation and augment the proficiency of healthcare professionals [5]. Furthermore, as highlighted by Darrudi A. et al. (2020), substandard care quality, coupled with inadequately regulated and fragmented healthcare delivery systems, escalating unmet health requirements, and the swift commercialization of healthcare within privatized systems constitute a significant challenge encountered by nations in their pursuit of universal health coverage [6]. As a result, there is a growing need to explore and use innovative approaches to improve and deliver safe, effective, and high-quality healthcare services in the healthcare system [7, 8].

The positive deviance approach represents an innovative strategy aimed at identifying exemplary practices that are present within a given community [9]. This community in the context of healthcare encompasses various entities, including teams, groups, departments, and organizations [10]. In this regard, departments and organizations could include regional/provincial, zonal, and district-based administrative health offices, local health facilities, and their respective units. For instance, social service agencies, representatives of health care organizations, and local government bodies have been considered as communities in an article that utilized PD to understand how social service and health care providers collaborate in communities that attain relatively low levels of health care utilization and expenses among senior citizens [11]. The approach recognizes the value of existing expertise [12] and operates on the premise that solutions to problems already exist within the community, and the act of identifying and disseminating these solutions can assist others in addressing existing complex and intractable challenges [9, 12]. Jason Gordon (2022) further emphasizes that despite having similar resource constraints as everyone else, there are individuals who excel in their performance to rules concerning organizational issues, and when given the opportunity, these PDs are willing to share their experiences as far as leaders facilitate the process [13]. Positive deviance has been applied in different sectors including social sciences, psychology, and healthcare [14], and yielded successful results in enhancing the nutritional status of children in multiple countries, such as Haiti, Vietnam, Pakistan, and India [15] and in preventing and addressing undernutrition and overweight among the adult population [15–17].

Application of PD is a somewhat lengthy process and involves different stages. The Bradley EH et al.‘s (2009) framework, which articulates a four-stage process, and the 4Ds/6Ds framework are commonly utilized in healthcare service-related articles. According to Bradley et al.‘s framework, the initial phase involves leveraging routinely gathered data to pinpoint organizations exemplifying exceptional performance, which is then subject to qualitative examination to formulate hypotheses regarding their superior outcomes. Subsequent stages involve the empirical validation of these hypotheses through statistical analysis in broader organizational samples and the dissemination of findings in collaboration with pertinent stakeholders to promote the replication of these best practices in comparable contexts [18]. The 4Ds/6Ds framework similarly starts with defining the issue at hand. In this framework, the first D stands for defining the problem and the next is for determining the presence of PDs or identifying them. The third D stands for discovering the successful but uncommon strategies that PDs apply in their practice and the fourth D represents the designing stage of interventions to allow others to apply these strategies in their practice. The final two Ds focus on evaluating the interventions’ effectiveness and ensuring the diffusion of effective practices to other entities [10, 19].

However, the available evidence on PD is mixed and there are controversies on the effectiveness of the approach, particularly in complex and demanding settings including the healthcare system. This is due in large part to methodological limitations of the extant studies including inconsistencies in the quality of strategies employed to identify PDs or positive deviant practices and the challenges related to the approach’s applicability in practical situations. For instance, a systematic review paper has reported that studies that applied the PD approach for quality improvement in complex interventions lack methodological quality and details in their work. The authors further stated that utilizing PD in healthcare settings poses challenges citing engaging staff more broadly in quality enhancement programs as a known difficult aspect [20]. As such, its potential for future use in the healthcare system is not well documented. Therefore, this scoping review aimed to synthesize the evidence regarding the use of the PD approach in healthcare system service delivery and quality improvement programs.

## Methods

This scoping review is conducted by using the Preferred Reporting Items for Systematic Reviews and Meta-analysis extension for Scoping Reviews checklist (PRISMA-ScR), as a guide for article screening, extraction, analysis, and presentation of the results [21]. In addition, the Population, Concept, and Context (PCC) framework was employed to assess the research question’s appropriateness and make amendments. Regarding article search, several international databases including PubMed, Embase, and Scopus were explored to retrieve studies related to PD and its application in the healthcare system. A complete search strategy was developed for the different databases and has been included as a supplementary file in this scoping review (see Additional file 1). Details regarding the article search strategy and further clarification of concepts including the PCC framework are available somewhere else [22].

### Research questions

The research questions of this scoping review are: (1) What research designs and methods are utilized in implementing PD in the healthcare system service delivery and quality improvement interventions?; (2) What are the strategies applied to identify PDs or measure positively deviant practices?; (3) What outcomes have been achieved using the PD approach?; and (4) What are the limitations in utilization of the PD approach, and what is its potential for use in the healthcare system service delivery and quality improvement programs?

### Eligibility, screening, and data extraction

Articles published only in the English language are included due to feasibility issues, and letters or comments to the editor, commentator, and brief communication articles and literature review studies other than systematic review and meta-analysis articles are excluded due to the nature of the scoping review detailed methodological requirements. In addition, only studies that described the methods used to assess the performance of PDs were included. Furthermore, all articles were assessed independently by two authors for inclusion through a title and, or abstract read, and those that passed the title and abstract review were screened by reading the full texts. Details are available elsewhere [22].

### Data analysis and presentation

A descriptive mapping and synthesis of the literature are employed to present data in text, table, and figure formats by using three major themes: Methodologies employed in using the PD including the strategies researchers utilized to identify PDs; outcomes or achievements reported from the usage of PD approach; and the prospects of PD in future health service research works. In addition, the results of this scoping review are reported based on the PRISMA-ScR guideline, and the entire process of study screening, selection, and inclusion is shown with the support of the PRISMA-ScR flow diagram [21].

## Results

### Characteristics of included studies

**Fig. 1:**
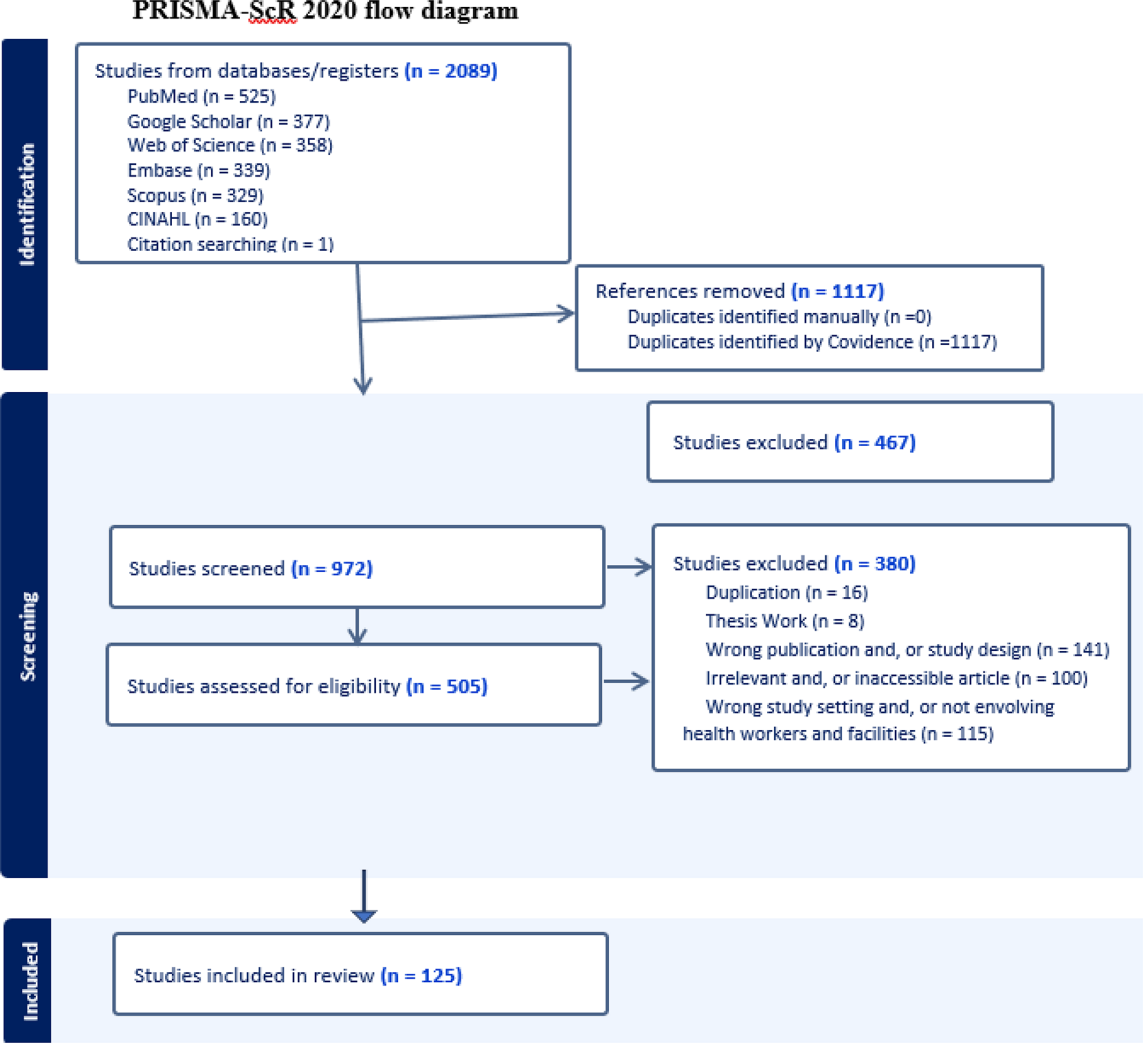
PRISMA-ScR flow diagram for the article selection process

We identified 2089 articles from different sources: PubMed (*n* = 525), Google Scholar (*n* = 377), Web of Science (*n* = 358), Embase (*n* = 339), Scopus (*n* = 329), CINAHL (*n* = 160), and reference searching (*n* = 1). Then, 125 articles were included for the scoping review after the title, abstract, and full-text screening, and removal of duplications (Fig. 1). Most of the included articles, 121 (96.8%) are original articles, and the rest, 4 (3.2%) are systematic reviews. Furthermore, more than half, 66 (52.8%) of the articles were from the United States, 11(8.8%) from multinational studies, 10 (8%) from Canada, 8 (6.4%) from the United Kingdom, 4 (3.2%) from Brazil, 4 (3.2%) from Israel, and the remaining, 22 (17.6%) are from other nations around the world (Table 1).

**Table 1:** Distribution of the included articles by country/countries (*n* = 125)

Country of Study	Frequency	Percent	Citation
Bosnia and Herzegovina	2	1.6	[23, 24]
Brazil	4	3.2	[25–28]
Canada	10	8	[29–38]
Colombia	1	0.8	[39]
Democratic Republic of Congo	1	0.8	[40]
Denmark	2	1.6	[41, 42]
Ethiopia	2	1.6	[43, 44]
Germany	2	1.6	[45, 46]
Indonesia	2	1.6	[47, 48]
Israel	4	3.2	[49–52]
Italy	1	0.8	[53]
Multinational	11	8.8	[20, 54–63]
Nepal	2	1.6	[64, 65]
Netherlands	1	0.8	[66]
Norway	1	0.8	[67]
Poland	1	0.8	[68]
Tanzania	1	0.8	[69]
Uganda	2	1.6	[70, 71]
United Kingdom	8	6.4	[72–79]
United States	66	52.8	[11, 80–144]
Vietnam	1	0.8	[145]
Total	125	100.0	125

### Methods followed in the utilization of PD for the healthcare system

#### Study design

Regarding the methodological composition of the research corpus, it was found that qualitative methods predominate, constituting 44.0% of study designs, followed by mixed methods approaches at 20.8%, pre-post intervention studies at 12.8%, and cross-sectional analyses at 12.0%. Notably, the foundational framework employed in most of the scrutinized articles is delineated in Bradley EH et al.‘s (2009) publication, which articulates a four-stage process [18], and the 4Ds/6Ds framework [10]. However, the literature exhibits a lack of uniformity in documenting the specific stages of PD examined or employed, and the temporal span of the studies encompasses the period from 2006 to 2023 (Supplementary Table 1).

#### Strategies utilized by researchers to identify PDs

In this scoping review, we found that the selection process of PDs is a difficult job, and no consistent validated criteria are utilized. Recommendation-based criteria have been utilized to identify PDs in twelve papers even though some of the criteria for selection are less clear and subject to potential bias [25, 34, 49, 52, 58, 79, 89, 94, 98, 106, 120, 123]. For instance, Toscos T, et al. (2018) used clinic liaison recommendations to identify positive deviant patients who displayed successful methods or practices for gaining access to healthcare, as well as healthcare workers who had developed successful care-delivery practices [94]. In addition, a combination of media influence and stakeholder recommendation [64], implementation-based [29], and experience-based criteria have also been used [31, 91]. In six articles, the criteria for the selection of PDs were unclear or unspecified [39, 47, 50, 76, 143, 145] and in the remaining papers, the selection of PDs does not apply as they are systematic reviews [20, 57, 59, 63] and adoption program papers that are implemented based on best practices identified from other positive deviant practices [28, 51, 54, 67, 92, 119]. However, performance-based criteria have been used to identify PDs at individual, health facility or health system levels in majority of the articles [11, 23, 24, 26, 27, 30, 32, 33, 35–38, 40–46, 48, 53, 55, 56, 60–62, 65, 66, 68–75, 77, 78, 80–88, 90, 93, 95–97, 99–105, 107–118, 121, 122, 124–142, 144].

### Outcomes reported from the utilization of PD

We found that significant outcomes have been reported in various domains from utilizing the PD approach for healthcare service and quality improvement research and interventions around the world. In some situations, researchers used the PD approach to identify successful strategies of PDs and build conceptual models for best practice. For example, Rakic S, et al. (2021) used the PD approach to identify the strategies top-performing health centers utilized for better financial sustainability in the Republic of Srpska, Bosnia and Herzegovina, and built an organizational-level model for public primary healthcare centers [24]. Similarly, Assefa Y, et al. (2014) employed the same approach to explore best practices that contributed to patient retention in HIV care and developed a framework for improving patient retention in future implementation programs [43]. In other cases, most of the included articles are conducted to identify PDs and the strategies that make PDs different from others at the health system, health facility, and, or individual levels. For instance, Klaiman TA, et al. (2014) utilized a PD framework to identify PD local health departments that had exceptional maternal and child health outcomes in the community using uniquely detailed and matched annual maternal and child health-related county-level expenditure data for all local health departments in Florida and Washington, USA [83].

#### Effectiveness in reducing medication errors and hospitalizations

Positive deviance is found to be an effective strategy in reducing medication errors and hospitalizations. In this regard, a study has indicated that in a 3-phase PD interventional program, using the approach was effective in reducing medication errors with each intervention resulting in a 0.12% decline rate in reported errors [28]. In addition, effectiveness in time management and financial expenditure, improved health outcomes, enhanced healthcare service quality, improved hand hygiene practice, reduction of infections, and reductions in adverse events after operation have been reported as indicators of success in utilizing PD in the healthcare system. For example, Tanenbaum J, et al. (2018) reported the evaluation of an adopted PD program in Ohio, USA, from a nationwide primary care–led regional quality improvement collaborative operating with hospitalization rates for ambulatory care sensitive conditions known as “Better Health Partnership” resulted in reductions in age- and sex-adjusted hospitalization rates in targeted ambulatory care sensitive conditions (diabetes, heart failure, and hypertension) in Cuyahoga County more than the rates in the comparator counties in 2009–11 (106 fewer hospitalizations per 100,000 adults) and 2012–14 (91 fewer hospitalizations) [143].

#### Effectiveness in time management and financial expenditure

Utilizing the PD approach in healthcare safety and quality programs has led to huge time and cost savings. For instance, Gold R, et al. (2023) revealed that the application of the PD approach has resulted in great improvements in time management in surgical cases across all time intervals of procedures with an overall time of 49.84 min saved per day compared to the baseline values [32]. Similarly, an improvement in operating room time management after successive value improvement initiatives has also been reported [30]. In addition, a reduction from 4.0 to 3.0 days in median length of stay after lobectomy in thoracic surgery following local PD seminars and from 4.0 to 3.5 days after multicentre seminars are reported as successes in surgical practice together with trends of decline in multiple adverse event rates [34]. Furthermore, the Ohio study cited above has also indicated that the decline in hospitalization rates in targeted ambulatory care sensitive cases was estimated to be 5,746 hospitalizations in 2009-14, resulting in nearly $40 million in cost savings [143]. In other programs, disposable materials cost reduction of $397.53 per lobectomy in thoracic surgery after cost awareness and surgeon engaging PD interventions with futures of sustainability [30], and an additional estimated $2-2.5 M revenue per year increment from saving time, without affecting quality, safety, and workload, after an introduction of PD strategy-based surgical prepping time protocol had been reported [113].

#### Improved hand hygiene practice and infection prevention

Several PD-based interventional studies have indicated that utilizing the approach has resulted in better healthcare service quality practices, including an increase in hand hygiene compliance rates [25, 26, 36, 61, 63], and a reduction of infections [25–27, 39, 61, 63, 92, 98, 106, 119]. Marra AR. et al. (2013) have revealed that improving hand hygiene practice in multiple inpatient settings has led to a decrease in the incidence of device-related healthcare-associated infections and the median length of stay after a PD intervention [61]. As an additional example, a monthly time series drop of healthcare-associated infection rates from 4.8 to 2.8 per 1000 patient-days [106], an increase in hand hygiene compliance, and a decline in Methicillin-resistant staphylococcus infection (MERSA) rates are also reported [63]. Furthermore, a sustained decreasing trend in the rate of device-related infections as a result of increasing use of alcoholic hand rub in nursing visits over 2 years [26], and a sharp decline in all access-related bloodstream infections from 2.04 per 100 patient-months preintervention to 0.75 after employing collaborative interventions and to 0.24 after augmenting the interventions with PD are documented in different settings [119]. Similarly, one program has reported several MRSA-free months in sustained and lasting changes in MERSA reduction rates, in the period after the application of PD without investing additional costs for the intervention [39].

#### Improved surgical care quality and reduction of complications

A trend in the reduction of multiple adverse event rates and health facility length of stay after pulmonary resection following best evidence- and best experience-based quality improvement PD seminars has been documented. In addition to the positive outcomes, the generation of universally applicable and successful consensus recommendations is made by the program for surgical safety and quality services [34]. Similarly, a reduction in post-operation complications after the application of surgeon self-evaluation in combination with a seminar-based PD quality improvement program has been observed in patients following non-cardiac thoracic surgery with a 34% decline in atrial fibrillation rates, a 38% decline in prolonged air leak rates and a 25% drop in anastomotic leak rates [34, 37].

#### Improved performance and care quality

Improvements in the quality of healthcare services and health outcomes have also been reported in different topics. For example, an overall increase in the proportion of controlled blood pressure (< 140/90 mmHg) among adult patients from 67% in 2013 to 74% in 2017) with improvements across all patient demographic and insurance subgroup types has been reported after implementation of a primary care practice regional health improvement collaborative programs in the USA [111]. In another interventional program, a consistent increase in the use of dual-method contraceptives was found among Ugandan women [70]. Similarly, PD clinician-owned practices have also been found to be more likely to achieve improvements in cardiovascular quality service outcomes without increasing health workers’ burnout than were practices owned by a hospital or health system [114]. Related to this, several strategies have also been reported to be significantly associated with lower risk-standardized mortality rates among patients with acute myocardial infarction in hospitals that implemented positive deviant practices in the USA. According to the report, not cross-training nurses from intensive care units for the cardiac catheterization laboratory, having cardiologists always on-site, holding monthly meetings for reviewing acute myocardial infarction cases between hospital clinicians and staff who transported patients to the hospital, fostering an organizational environment in which clinicians are encouraged to solve problems creatively, and having physician and nurse champions rather than nurse champions alone resulted in 0.44, 0.54, 0.70, 0.84 and 0.88% point reductions in a 30-day risk-standardized mortality rate respectively [87]. Moreover, a mental health support group program facilitated by Women’s Union staff for people with severe mental illness in rural Vietnam has indicated that the use of the PD approach was associated with a substantial improvement in personal functioning and reduction of stigma and discrimination among patients. In addition, the authors have reported that the intervention has resulted in a significant reduction in financial burdens for the families of patients with severe mental illness, and the program was feasible and highly acceptable by the community [145].

### Reported limitations/challenges in using PD

The included articles have mentioned several limitations that can be related to the PD approach even though it is not possible to prove whether it is true or not as many of the limitations can be related to the specific study design of individual articles. Some examples of commonly reported limitations included are thematized and cited below as concerns in operationalizing who positive deviant is or are [55, 57, 66, 74, 95, 101, 107], the difficulty of confirming true PDs in comparison with others, as a change in time may affect outcomes and PDs are commonly identified with secondary data, and, or stored data in the past [83, 85, 102, 107, 115, 143] a small number of participants [31, 41, 91, 107], focus on leaders and senior managers and limited staff and patient, or other stakeholder perspective involvement [11, 41, 64, 72, 81, 82, 87, 96], response bias including social desirability bias [55, 64, 81, 84–87, 96, 101, 102], and issue of not achieving information saturation in qualitative studies as PD is mainly qualitative in its initial stages [82].

Furthermore, limited study setting/s [30, 31, 41, 44, 80, 82, 88, 90, 101], difficulty of performing statistical tests due to small sample size [33, 65, 83], difficulty of adjusting confounders [30, 34, 63, 68, 114], lack of comparison and, or difficulty of doing comparative analysis [24, 71, 82], and issues of generalizability/transferability [11, 30, 42, 44, 64, 72, 80, 82, 84, 91, 96, 98, 100, 102, 143] are also reported as common limitations in several articles. In addition, the none-probability sampling methods used to recruit participants [94] and the Hawthorne effect, a type of reactivity in which individuals modify an aspect of their behavior in response to their awareness of being observed as positive deviant are included in many articles as limitations in using the approach of PD [30, 32, 49, 66, 71]. These limitations highlight the need for careful consideration of a balanced method when using the PD approach [146].

## Discussion

The overall uptake and publishing of PD studies for health care service and quality of care improvement is quite limited and heavily USA-focused. This distribution is unexpected as the PD approach is primarily believed to have huge significance for resource-limited settings [20]. The reason for this could be because the PD approach is an emerging science and may not be adequately promoted in many low- and middle-income countries. In addition, from the methodology perspective, the scoping review findings have indicated that diverse types of study designs can be applied in utilizing the PD approach for healthcare service and quality improvement programs. This could be surprising for some people because, during the initial stages of the review protocol development, some of the author’s understanding was that only limited study designs could be applicable in using the PD approach as the method utilized in identifying the strategies of PDs is mainly qualitative [18]. However, in line with our anticipation, most of the included articles utilized qualitative, mixed, and pre-post interventional designs. These are useful in utilizing PD and identifying the strategies of top performers in comparison with others. This is because, qualitative research is useful to understand the “why” behind the strategies and behaviors of top performers and uncover the motivations, challenges, and contextual factors that contribute to their success, and the mixed cross-sectional and interventional methods research can combine quantitative data to quantify the prevalence and impact of certain strategies, reinforce qualitative findings and offer a more comprehensive picture [147].

Furthermore, pre-post interventional designs are important to evaluate how the strategies implemented by top performers lead to improvements over time and in comparison with others who do not implement these strategies [148, 149]. An example of this is the study conducted by Curry LA, et al. (2018) in 10 hospitals in the USA to enhance improvements in different domains of organizational culture that lead to significant changes in culture between baseline and 24 months, especially with regards to the learning environment and senior management support. The study further added that six hospitals having major cultural transformations reported considerably higher reductions in risk-standardised mortality rates in patients with acute myocardial infarction than four hospitals that did not shift culture citing a 1.07 and 0.23% point changes in the risk-standardised mortality rates between the two groups respectively over the period of 2011–2014 and 2012–2015 [150]. These findings are supported by studies from other disciplines as well. For instance, a systematic review paper has indicated that 21 randomized controlled trial studies were utilized to improve employee well-being and effectiveness using web-based psychological interventions delivered in the workplace [151]. In addition, another systematic review paper has indicated that qualitative studies, pre-and post-test design without a control, non-randomized trials, non-randomized cross-sectional studies, and randomized controlled trials were utilized in community-based nutritional programs conducted based on the PD approach to reduce childhood malnutrition across the globe [17].

Concerning the identification of PDs, the selection process has been reported to be a difficult task in the utilization of PD [20]. The scoping review findings have supported this statement and depicted that the selection process of PDs appeared to be too complex and dependent on the unique circumstances of each study or project. As a result, different articles and studies have utilized a range of methods and criteria to identify PDs including peer recommendations [25, 49, 52, 106, 120, 123], and performance-based criteria such as lowest risk-adjusted morbidity [139], mortality [82, 87, 93, 102], timely service [26, 32, 33, 84, 95, 99, 113, 116, 134], and standardized composite performance measures including high composite quality scores [86, 115, 137], high composite performance/healthcare service scores [11, 23, 27, 43–45, 53, 62, 65, 69, 75, 78, 109, 110, 117, 118, 122, 124, 131, 138, 140, 144], and better clinical outcome scores [37, 38, 111, 118, 125, 130]. This justifies the absence of a one-size-fits-all approach to selecting PDs, and the criteria used can vary greatly based on the specific domain and goals of initiatives. However, the use of non-standardized and, or non-validated criteria can lead to bias and this in turn can lead to failure in identifying true PDs [55, 57, 66, 74, 95, 101, 107]. For instance, recommendation-based criteria can still be used to identify positive deviants in settings where there is no sufficient resource and data to identify positive deviant practices. However, the researchers need to be cautious of possible bias in the process of identifying true positive deviants when using such methods and need to ensure whether the recommenders are genuine or not and use other performance-based rating criteria in other situations when using validated and, or standardized tools are applicable.

In line with the above findings, another scoping review paper has indicated that some researchers have used unstandardized and less clear criteria to select PDs and suggested that objective measures of health outcomes should be used whenever possible to reduce the possibility of bias in examining associations [146]. Furthermore, it is critical to consider the underlying assumption that the identified PDs are contextually comparable with other groups in terms of access to resources and expertise [20]. This is because, health workers, health facilities, and/or health systems with better expertise and financial resources could have advantages that can influence their performance and the subsequent outcomes in comparison with their counterparts signifying the need to consider the degree of resources between populations or groups when designing a sampling strategy [20, 146]. This challenge can be addressed to some extent in the selection criteria. For instance, in one study, high and low-performing hospitals were purposefully selected from within a random set of top 5% and bottom 5% ranked hospitals to ensure diversity in areas such as volume of patients with acute myocardial infarction, teaching status, and socioeconomic status of patients [152].

Regarding the reported outcomes, the PD approach has been employed for several aims and objectives. For instance, Klaiman TA, et al. (2014) utilized a PD framework to identify positive deviant local health departments that had exceptional maternal and child health outcomes in the community. The authors used uniquely detailed and matched annual maternal and child health-related county-level expenditure data for all local health departments in Florida and Washington. The data were sourced from the Public Health Activities and Services Tracking database for identifying high-performing local health departments. These data were linked with factors depicting the local context and local health department structures [83]. In other cases, many of the articles are conducted to identify the strategies of PDs that make them successful and share their experience with the wider community. For example, Borghini A, et al. (2021), employed a mixed-methods study to identify PDs regarding maternal care at the regional level in 10 Italian regions. In the first phase, the researchers used quantitative data including performance metrics and maternity care indicators to identify top-performing regions. In the second phase, they investigated the organizational determinants and the experience of healthcare workers involved in the process qualitatively and identified seven best practices including the existence of trust among healthcare professionals and having shared goals as the reasons for better performance and success in comparison with other regions [53].

Furthermore, researchers have proved that the utilization of PD has helped them be successful in various healthcare services and quality improvement programs including in the reduction of medication errors [28], enhancing effectiveness in time management and/or financial expenditure [30, 32, 143], in improving hand hygiene practice and, or reducing infection rates [26, 27, 36, 39, 51, 61, 63, 92, 98, 106, 119], in improving surgical care quality and reduction of complications [34, 37], and in other reported outcome measures including an increase in the proportion of controlled blood pressure (< 140/90 mmHg) among adult patients in the USA [111], a consistent increase in the use of dual-method contraceptives among women in Uganda [70], improvement in cardiovascular quality service outcomes [114], and improvement in personal functioning’s and reduction of stigma and discriminations among patients with severe mental illnesses after implementation of PD-based programs have indicated the huge potential of utilizing PD in healthcare service quality and other outcome improvement efforts [145]. These successes from the utilization of the PD approach are supported by other studies from different programs. For instance, a systematic review paper has indicated that the utilization of the PD approach has helped countries to reduce childhood malnutrition across the globe [17].

Similarly, a 65–80% persistent decline in childhood malnutrition in Vietnam and a substantial reduction in other communities across 41 different nations around the world, in programs that used PD has also been reported [8, 153]. In addition, success in the prevention and treatment of undernutrition, overweight, and obesity in socioeconomically vulnerable mothers and the adult population has also been reported [15, 16]. Furthermore, a reduction in neonatal mortality has been reported in Pakistan [8, 153]. This could be because the strategies of PDs are internally driven and the solutions are generated within the community that can be implemented with minimal expenditures, particularly in settings with financial hardship [8, 57, 154]. However, it is important to note that each healthcare context is unique, and the success of PD interventions may depend on factors such as the specific challenges being addressed, the engagement of stakeholders, and the adaptability of the interventions to the local context [18, 154].

From the prospect’s perspective, several limitations have been mentioned in the primary articles including issues in the operationalization of PD, the difficulty of identifying true PDs during selection time because of time-related changes in practice about who positive deviant is or are, bias, and issues in generalizability/transferability of results. Majority of these limitations might be specific to the respective studies design, setting, and contexts [155, 156]. However, the cited limitations could have been attributed not only to the nature of specific studies but also to the peculiar nature of the PD approach. This is because the PD approach has assumptions and principles that need to be followed when selecting study participants whether it is at individual, group, or organization level. For instance, Bradley EH, et al.’s (2009) 4-stage framework that is proposed to be followed for healthcare quality research works in using the PD approach suggests using routinely collected data in identifying positive deviant organizations that consistently exhibit exceptionally high performance in the area of interest at stage 1, intensively examine the organizations using qualitative methods to generate hypotheses about practices that help organizations achieve top performance at stage 2; statistically test hypotheses in larger, representative samples of organizations at stage 3 and collaborate with key stakeholders to disseminate the findings, such as the organizations’ customers and suppliers at stage 4 [18].

As a result, when studies are conducted, following those assumptions can pose a limitation on the research designs selected and procedures followed including selection of PDs and using routinely collected data in measuring performance may affect the results of studies as the practice level is dynamic and progressive over time. For instance, Curry LA, et al. (2011) cited that study sites (hospitals) were visited at a single point in time, and using secondary data might not be good for identifying true PDs as their performance could have been changing (improving or declining) and others, in contrast, could be top performing at the time of applying PD [85]. The possible reason for this is that the continuous nature of practice improvement over time could affect the selection of positive deviant sites as other comparators may progress as well and top performers might become low-performing at another time [85, 102]. This highlights the need to use robust criteria in the selection process of PDs [146].

Concerning the potentials, utilization of PD not only has been successfully employed in healthcare service and quality improvement programs but also the variables included as limitations or challenges in the articles are potentially manageable. For instance, the issue of operationalization of PD can be reduced or solved by using risk-adjusted and standardized composite measures including utilization of validated performance-based criteria and considering the consistency of top-performing individuals, healthcare organizations, and systems background history as one of the assumptions of PD is the consistency of performance over time [20]. In addition, combining the evaluation of the current practice level of PDs with their historical data in comparison with other assumed low performers can be a solution for these issues and reduce the effect of the changing and progressive nature of practice between high-performing and low-performing individuals, teams, and, or healthcare facilities. Furthermore, using data that can show consistent performance for several years could be one possible option that can reduce the effect of time change in using such data sources [18]. For instance, the Curry et al. (2011) study selected hospitals that showed consistency and ranked in either the top or the bottom 5% in risk-standardized mortality rates among patients with acute myocardial infarction over two 2 years. This was seemingly intentionally done to mitigate the effect of time change on the performance level of health facilities and identify the hospitals that are truly positively deviant in comparison to those that are negatively deviant [85]. In addition, several of the included studies have used validated criteria to identify PDs that can support the above findings including lowest risk-adjusted morbidity [139], mortality [82, 87, 93, 102], lowest age-adjusted mortality [136], and standardized composite performance measures including high composite quality score [86, 115, 137], and high composite performance/healthcare service scores as using row morbidity and mortality rates without confounder adjustment is a non-reliable indicator of high and low performance [11, 23, 27, 43–45, 53, 62, 65, 69, 75, 78, 109, 110, 117, 118, 122, 124, 131, 138, 140, 144, 157].

Limitation of generalizability of results has also been mentioned related to small sample size, limited study setting, study design, and non-probability sampling techniques [11, 30, 42, 44, 64, 72, 80, 82, 84, 91, 96, 98, 100, 102, 143] and, or due to other peculiar nature of included studies as PD approach assumes exceptional performance that is quite different from the norm even with the inclusion of comparators [8, 18]. However, it is worth considering that findings from qualitative studies are not intended to be generalized, but rather to provide insights into previously unexplored areas and to generate hypotheses for future quantitative evaluation [158]. In PD, qualitative studies help researchers to identify best practices from high achievers that can be adopted in other settings with similar contexts. Therefore, the results of PD-related studies may not be generalizable to other settings due to their intentional focus on high-performing teams or organization/s and usage of qualitative research designs as its foundation [18]. In addition, the issue of not testing statistical relationships [33, 65, 83] might not be an issue as the fundamental principle of qualitative studies is to generate ideas from a small sample of the population; therefore, the results can be tested in a larger and more representative sample of the population. This signifies the need to conduct a quantitative study after developing a testable hypothesis from the qualitative studies depending on the nature of their research questions due to the need for research findings to have a statistically significant relationship if it is to be scientifically accepted as a predictor variable or variable associated with another outcome variable [18]. Above all, it is paramount to consider that even in qualitative studies, the results can still apply to other contextually similar situations as far as rigorous procedures are followed by authors in conducting their qualitative studies. This is known as theoretical generalizability or transferability [159].

Moreover, limited study setting [30, 31, 41, 44, 80, 82, 88, 90, 101], and limited staff involvement are also repetitively mentioned as a challenge in utilizing PD [11, 41, 64, 72, 81, 82, 87, 96]. This has been reflected in many studies suggesting that the process of recruiting study participants might disproportionately focus on leaders and senior managers, potentially neglecting insights from frontline staff who also contribute to successful practices. Related to this, the issue of possible failure to achieve information saturation has also been included [82]. This challenge should be addressed via the use of robust qualitative methods that if done well could ensure both a broad sample of respondents (especially the front line) and employ saturation as a criterion for sample size and data collection iteration. This justification has been supported by the fact that several other studies have considered multiple settings and stakeholders including frontline staff in their studies signifying the importance of taking multiple participants from various settings and including different stakeholders if researchers are to get rich sources of information on the performance of health facilities and other responsible bodies within the health system [78, 81, 84, 91, 95, 110, 112].

Furthermore, although lack of comparison has been mentioned as a limitation in some studies, the inclusion of comparators in the implementation of the PD approach has been mentioned as a strength as well and proved its importance in testing the impact of PD-based interventional studies [25–28, 32, 34, 36, 37, 39, 61, 70, 98, 106, 113, 119, 143, 145]. The reason why it is important to include comparators in the utilization of PD is that it helps researchers to uncover the traits that distinguish high performers from low performers, as well as to shed light on the unproductive practices of low performers. In addition, performing statistical tests by including comparators is also recommended if the later stages are to be implemented after the identification of PDs, as in stage 3 of Bradly EH, et al.’s (2009) framework [18]. Furthermore, in implementation research, a PD intervention can be applied to one group, and the results can be compared to a control group that was not exposed to the PD intervention, allowing researchers to investigate the true impact of the positive deviant practice/s in other similar settings [160]. However, this may not be always possible due to various reasons including refusal of low-performing individuals, groups, and, or healthcare settings due to fearing reprisal and other reasons. Moreover, resource constraints may make the inclusion of comparators very challenging in different situations. In such instances, other options can be considered, if comparison is needed including before and after intervention analysis as pre-post interventional designs play a crucial role in assessing the efficacy of strategies employed by high achievers in other similar settings, elucidating their impact over time [148, 149].

Different forms of bias including recall, social desirability, interviewer and researcher biases, and the Hawthorne Effect have been also cited as limitations in many articles [55, 64, 81, 84–87, 96, 101, 102]. However, even though the approach has a special focus on qualitative studies, these issues are not specific to PD and can be handled contextually. For instance, blinding of study participants, data collectors, and analyzers can be done to reduce the effect of social desirability, interviewer, and researcher biases, respectively. This is because even though the PD approach commands the researchers to identify top-performing individuals, groups, and organizations, and identify their strategies for being successful [18], the data collectors, study participants, and data analyzers can still be blinded regarding the research design and who positive deviant is and is not. An example of bias in data analysis is a faulty interpretation. This occurs, when authors approach analysis intending to justify their belief or perspective, which can potentially lead to the discovery of facts that support their point of view invariably [161]. Therefore, having different data analyzers and comparing the results of the outcomes could help in reducing such biases. In addition, blinding study participants about what they are going to be asked may not be applicable, in fact, however, at least they still can be blinded to whether they or their facility is considered as positive deviant or not. Recall and social desirability biases and the Hawthorne Effect would also likely act similarly among participants from positive and negative deviant categories, limiting its impact on the comparisons provided, particularly, if the participants did not know their deviant category, suggesting the importance of blinding high and low performing classifications [115].

Nevertheless, as with other approaches, different strategies can be employed to minimize the effect of bias in the utilization of PD. For instance, one article used three techniques to minimize the effect of researchers’ preconceived biases from affecting the results. These techniques were (1) enhancing the attention of researchers’ reflexiveness through systematic debriefings with an organizational psychologist; (2) utilizing a multidisciplinary team to analyze transcripts critically with an explicit focus on identifying negative (disconfirming) cases; and (3) training of interviewers on the strategies of data collection including how to encourage participants to get both reliable data during interviews, without interviewer judgment [82]. In summary, incorporating different strategies can enhance the robustness and applicability of PD-related studies in healthcare and other fields. As a result, careful planning and addressing potential design flaws and methodological limitations are important for deriving meaningful conclusions from such studies [10, 18].

### Implications

Several articles have proved the successfulness of utilizing the PD approach in multiple areas of the healthcare system including in improving quality of care, increasing hand hygiene compliance rate, infection prevention, prevention of postoperative complications, and medication errors. This indicates the enormous potential of utilizing the PD approach for healthcare service quality improvement programs. Moreover, even though some earlier articles stated that the utility of the PD approach is limited in complex settings including the healthcare system [20], and various included articles have mentioned several limitations, as highlighted before, these issues are potentially manageable if they are carefully handled [10, 18].

### Strength and limitations

This scoping review only included articles that are conducted at the health system, health facility, and individual levels within the healthcare systems. In addition, only studies published in the English language were considered. Another limitation is that some articles might be missed due to failing to use the phrase “positive deviance”, despite utilizing the approach in their studies, related to inconsistent use of different terminologies to describe PDs in several situations [57]. However, comprehensive searches were employed across multiple databases using predefined search strategies.

## Conclusion

Positive deviance has been extensively utilized for healthcare service and quality enhancement programs around the world, particularly in developed countries as most of the articles are from three countries (USA, Canada, and the United Kingdom) with the USA contributing to more than half of all the included articles. In addition, several types of study designs including qualitative, mixed-methods, interventional, and randomized controlled trials have been employed by researchers and program implementers depending on the context and specific objectives of their project works. By identifying and learning from those who exhibit exceptional practices, healthcare systems have been able to implement successful strategies, enhance quality, reduce errors, and improve patient outcomes. However, all the outcomes of PD-based research depend on the first step of identifying true PDs. This is because unless studies use objective and validated measures of performance, identification of true PDs is less likely, and this can in turn result in failure to identify best practices for learning. Therefore, researchers need to take different contexts in the initial stages of PD into consideration to avoid the problems reported in the identification processes of PDs including the usage of standardized performance measures. In addition, the use of comparators and exploring the practice of low performers can help to uncover the traits that distinguish good performers from low performers, as well as to shed light on unproductive practices.

### Electronic supplementary material

Below is the link to the electronic supplementary material.

Supplementary Material 1

Supplementary Material 2

Supplementary Material 3

## Data Availability

Data is provided within the manuscript or supplementary information files.

## Abbreviations

PCC: Population Concept and Context
PRISMA-ScR: Preferred Reporting Items for Systematic Reviews and Meta-analysis extension for Scoping Reviews
PD: Positive Deviance
PDs: Positive Deviants
UHC: Universal Health Coverage
USA: United States of America
WHO: World Health Organization
