# Supplementary material for: The use of positive deviance approach to improve health service delivery and quality of care: a scoping review
Source: BMC Health Serv Res. 2024 Apr 8;24:438. doi: 10.1186/s12913-024-10850-2 (PMC11003118; doi:10.1186/s12913-024-10850-2)
Supplement: Supplementary file 1 — Supplementary Material 1 [file 12913_2024_10850_MOESM1_ESM.docx]

**Additional file 1: Search strategies applied for all major databases and number of articles retrieved in each database.**

1. **PubMed Search Strategy**
   1. *(("Positive Devian*" OR "Positive Deviance" OR "Deviant Approach") AND (Method* OR Procedure* OR Technique OR Strateg* OR Achieve* OR Success* OR Accomplishment* OR Attainment* OR Outcome* OR Prospect* OR Challenge* OR Limitation* OR Potential*)) AND (Health* OR Healthcare* OR "Healthcare*" OR "Health System*" OR "Health Service*" OR "Health Equity" OR "Quality Improvement*" OR "Health Behavior*" OR "Health Promotion*" OR Prevent* OR Treatment* OR "Palliative Care").*
   - **Combinations**: ("positive devian*"[All Fields] OR "Positive Deviance"[All Fields] OR (("deviant"[All Fields] OR "deviants"[All Fields]) AND ("approach"[All Fields] OR "approach s"[All Fields] OR "approachability"[All Fields] OR "approachable"[All Fields] OR "approache"[All Fields] OR "approached"[All Fields] OR "approaches"[All Fields] OR "approaching"[All Fields] OR "approachs"[All Fields]))) AND ("method*"[All Fields] OR "procedure*"[All Fields] OR ("methods"[MeSH Terms] OR "methods"[All Fields] OR "technique"[All Fields] OR "methods"[MeSH Subheading] OR "techniques"[All Fields] OR "technique s"[All Fields]) OR "strateg*"[All Fields] OR "achieve*"[All Fields] OR "success*"[All Fields] OR "accomplishment*"[All Fields] OR "attainment*"[All Fields] OR "outcome*"[All Fields] OR "prospect*"[All Fields] OR "challenge*"[All Fields] OR "limitation*"[All Fields] OR "potential*"[All Fields]) AND ("health*"[All Fields] OR "healthcare*"[All Fields] OR "healthcare*"[All Fields] OR "health system*"[All Fields] OR "health service*"[All Fields] OR "Health Equity"[All Fields] OR "quality improvement*"[All Fields] OR "health behavior*"[All Fields] OR "health promotion*"[All Fields] OR "prevent*"[All Fields] OR "treatment*"[All Fields] OR "Palliative Care"[All Fields]). **525 Results with all fields searches and without adding any filter.**
2. **Scopus search strategy**
   1. ( TITLE-ABS-KEY ( "Positive Devian*" OR "Positive Deviance" ) AND TITLE-ABS-KEY ( method* OR procedure* OR technique OR strateg* OR achieve* OR success* OR accomplishment* OR attainment* OR outcome* OR prospect* OR challenge* OR limitation* OR potential* ) AND TITLE-ABS-KEY ( health* OR healthcare* OR "Healthcare*" OR "Health System*" OR "Health Service*" OR "Health Equity" OR "Quality Improvement*" OR "Health Behavior*" OR "Health Promotion*" OR prevent* OR treatment* OR "Palliative Care" ) ). **Number of articles retrieved with title, abstract and Key word search:** **329 document results. Search date: June 2, 2023.**
3. **Embase Search Strategy**
   1. ('positive devian*' OR 'positive deviance'/exp OR 'positive deviance' OR 'deviant approach') AND (method*:ti,ab,kw OR procedure*:ti,ab,kw OR technique:ti,ab,kw OR strateg*:ti,ab,kw OR achieve*:ti,ab,kw OR success*:ti,ab,kw OR accomplishment*:ti,ab,kw OR attainment*:ti,ab,kw OR outcome*:ti,ab,kw OR prospect*:ti,ab,kw OR challenge*:ti,ab,kw OR limitation*:ti,ab,kw OR potential*:ti,ab,kw) AND (health*:ti,ab,kw OR healthcare*:ti,ab,kw OR 'healthcare*':ti,ab,kw OR 'health system*':ti,ab,kw OR 'health service*':ti,ab,kw OR 'health equity':ti,ab,kw OR 'quality improvement*':ti,ab,kw OR 'health behavior*':ti,ab,kw OR 'health promotion*':ti,ab,kw OR prevent*:ti,ab,kw OR treatment*:ti,ab,kw OR 'palliative care':ti,ab,kw). **Number of articles retrieved with title, abstract and Key word search:** **339 document results. Search date: June 2, 2023.**
4. **Web of Science Search Strategy**
   1. ("Positive Devian*" OR "Positive Deviance" OR "Deviant Approach") (All Fields) AND (Method* OR Procedure* OR Technique OR Strateg* OR Achieve* OR Success* OR Accomplishment* OR Attainment* OR Outcome* OR Prospect* OR Challenge* OR Limitation* OR Potential*) (All Fields) AND (Health* OR Healthcare* OR "Healthcare*" OR "Health System*" OR "Health Service*" OR "Health Equity" OR "Quality Improvement*" OR "Health Behavior*" OR "Health Promotion*" OR Prevent* OR Treatment* OR "Palliative Care") (All Fields). **Timespan:**1900-2023. **Results from Web of Science Core Collection: 358.** **Search date: June 2, 2023.**
5. **CINHAL Search Strategy**
   1. (("Positive Devian*" OR "Positive Deviance" OR "Deviant Approach")) AND ( (Method* OR Procedure* OR Technique OR Strateg* OR Achieve* OR Success* OR Accomplishment* OR Attainment* OR Outcome* OR Prospect* OR Challenge* OR Limitation* OR Potential*)) AND ((Health* OR Healthcare* OR "Healthcare*" OR "Health System*" OR "Health Service*" OR "Health Equity" OR "Quality Improvement*" OR "Health Behavior*" OR "Health Promotion*" OR Prevent* OR Treatment* OR "Palliative Care")). **Search mode:** **Default** **optional**. **Results**=160, **Search date: June 2, 2023.**
