# Supplementary material for: The use of positive deviance approach to improve health service delivery and quality of care: a scoping review
Source: BMC Health Serv Res. 2024 Apr 8;24:438. doi: 10.1186/s12913-024-10850-2 (PMC11003118; doi:10.1186/s12913-024-10850-2)
Supplement: Supplementary file 3 — Supplementary Material 3 [file 12913_2024_10850_MOESM3_ESM.docx]

**Supplementary table 1**. Distribution of articles by study design and publication year (n=125).

| **Study design** | | **Frequency** | **Percent** | |
| --- | --- | --- | --- | --- |
|  | A 3-round policy Delphi study | 1 | | 0.8 |
|  | Cohort study | 2 | | 1.6 |
|  | Cross-sectional study | 15 | | 12.0 |
|  | Mixed methods | 26 | | 20.8 |
|  | Not specified | 1 | | 0.8 |
|  | Pre-postintervention | 16 | | 12.8 |
|  | Prevalence study | 2 | | 1.6 |
|  | Qualitative research | 55 | | 44.0 |
|  | Randomized controlled trial | 3 | | 2.4 |
|  | Systematic review | 4 | | 3.2 |
|  | **Total** | **125** | | **100.0** |
|  | **Publication year** | **Frequency** | | **Percent** |
|  | 2006 | 1 | | 0.8 |
|  | 2008 | 2 | | 1.6 |
|  | 2009 | 1 | | 0.8 |
|  | 2011 | 3 | | 2.4 |
|  | 2012 | 3 | | 2.4 |
|  | 2013 | 9 | | 7.2 |
|  | 2014 | 6 | | 4.8 |
|  | 2015 | 4 | | 3.2 |
|  | 2016 | 9 | | 7.2 |
|  | 2017 | 8 | | 6.4 |
|  | 2018 | 16 | | 12.8 |
|  | 2019 | 8 | | 6.4 |
|  | 2020 | 16 | | 12.8 |
|  | 2021 | 15 | | 12.0 |
|  | 2022 | 10 | | 8.0 |
|  | 2023 | 14 | | 11.2 |
|  | **Total** | **125** | | **100.0** |

The data in the supplementary table shows the distribution of included articles in the scoping review by study design and publication year.
